# Regulatory T Cell Sub-Populations in Patients with Distinct Autoimmune/Inflammatory Diseases With or Without Inborn Errors of Immunity

**DOI:** 10.3390/diagnostics15151879

**Published:** 2025-07-26

**Authors:** Sevil Oskay Halacli, Dilan Inan, Saliha Esenboga, Hacer Neslihan Bildik, Aslihan Berra Bolat, Ilhan Tezcan, Deniz Cagdas

**Affiliations:** 1 Division of Pediatric Immunology, Department of Basic Sciences of Pediatrics, Institute of Child’s Health, Ankara 06230, Turkey; 2 Division of Pediatric Immunology, Department of Pediatrics, Faculty of Medicine, Hacettepe University, Ankara 06100, Turkey; 3 Translational Medicine Laboratories, Faculty of Medicine, Hacettepe University, Ankara 06100, Turkey; 4 Faculty of Medicine, Hacettepe University, Ankara 06100, Turkey

**Keywords:** regulatory T cells, sub-population, autoimmune disease, inflammatory disease, primary immunodeficiency

## Abstract

**Background**: Regulatory T cells (Tregs) are the main suppressor cells that maintain immune tolerance and prevent autoimmunity. Changes in Treg number or function are implicated in a wide range of autoimmune and inflammatory (AI/I) diseases, with or without underlying inborn errors of immunity (IEI). Understanding the phenotypic profiles of Treg subsets and their associations with immune dysregulation is crucial to identifying potential robust and holistic biomarkers for disease activity. **Methods**: We examined peripheral blood mononuclear cells from 40 patients diagnosed with various autoimmune/inflammatory diseases, including those with genetically confirmed inborn errors of immunity (IEIs), and compared these samples to those from 38 healthy controls of the same age. Utilizing multiparametric flow cytometry, we measured multiple Treg sub-populations and investigated their correlations with lymphocyte subset profiles and the diversity of autoantibodies. We applied advanced statistical and machine learning techniques, such as t-SNE, k-means clustering, and ROC analysis, to analyze immunophenotypic patterns in the patients. **Results**: Among all Treg sub-populations, only CD4^+^CD127^low^CD25^high^FOXP3^+^ Tregs showed a significant decrease in patients compared to healthy controls (*p* < 0.05), while other Treg phenotypes did not differ. FOXP3 expression showed reduced intensity in patients and demonstrated diagnostic potential (AUC = 0.754). Notably, this Treg subset negatively correlated with CD19^+^ B cell percentages and positively correlated with the diversity of circulating autoantibodies. Unsupervised clustering revealed three distinct immunophenotypic profiles, highlighting heterogeneity among patients and underlining FOXP3-centered immune dysregulation. **Conclusions**: Our results presented that patients have an impairment in the CD4^+^CD127^low^CD25^high^FOXP3^+^ regulatory T cell subset, which is identified by significantly decreased frequency and decreased expression of FOXP3. Immunological heterogeneity among patients was further uncovered by unsupervised clustering, highlighting the critical role that FOXP3-centered regulatory failure plays in the pathophysiology of illness. The combined evaluation of these three immunological factors, centered around FOXP3, holds promise as an integrative tool for monitoring disease progression across various autoimmune and immunodeficient contexts.

## 1. Introduction

A balanced cellular response of the adaptive immune system is responsible for eliminating the pathogenic antigens derived from bacteria, viruses, fungi, and malignantly transformed cells while preventing damage to self-tissues. AI/I disorders may occur when the excessive immune response affects the relevant tissues or organ systems due to a corrupted immune balance, which is called immune dysregulation [1,2]. Immune dysregulation is frequently represented with AI/I diseases in patients and may emerge from either primary factors which reveals inborn errors of immunity due to the mutations in *FOXP3*, *AIRE*, *LRBA*, *CTLA4* and *STAT1*, *STAT3* genes [3,4,5,6] or secondary causes such as infections by *Mycobacterium avium* species and *Streptococcus pyogenes* in inflammatory bowel disease [IBD] and acute rheumatic fever, respectively [7,8].

Regulatory immune cells are responsible for the equilibration of the immune response, and Tregs are well-known immune suppressor cells by inhibiting conventional T helper cells via cell-to-cell contacts with antigen-presenting cells or by secreting inhibitor cytokines such as IL-10, IL-35, and TGF-β [9,10]. Tregs are differentiated during the early gestational period in the thymus like other conventional CD4^+^ T helper cells and are named as thymic or natural Tregs [nTregs] or in the peripheral tissues such as skin and gut via microenvironmental cytokines [pTregs] [9,10]. In humans, different nTreg and pTreg subsets are characterized by their differential expression of specific surface markers, such as CD25 and CD127, as well as transcription factors, such as FOXP3. Considerable expression of CD25, which is a receptor for IL-2, is associated with the suppressive capacity of Tregs. In addition, the absence of CD127 expression is used as a complementary surface marker in the determination of Tregs. Expression of FOXP3 is considered a key transcription factor in the development and sustainability of the Tregs, even though some sub-populations of Tregs with suppressive effects do not express it [11,12,13,14,15,16,17]. Considering their differential expressions, different Treg sub-populations are investigated separately to associate with the suppressive aspect of the immune system in particular AI/I diseases in clinical diagnostic laboratories. Defects in the number, ratio, and function of Tregs are important for the development of AI/I diseases [18,19,20,21,22,23,24,25,26]. The numbers or ratio of different Treg sub-populations are analyzed in many of the AI/I diseases such as Multiple Sclerosis [MS], T2DM [Type 2 Diabetes Mellitus] and atopic dermatitis in samples derived from peripheral blood and relevant tissues of patients even though controversial results were obtained from different patients cohorts [27,28,29,30,31,32,33,34,35,36,37]. Decreased percentages of Treg sub-populations were observed in distinct autoimmune thyroid diseases, which have different pathophysiological mechanisms [26]. Another study that investigates Treg sub-populations to find a proper Treg marker in distinct rheumatological diseases with different immuno-pathophysiologic mechanisms to show the disease activity [29].

Based on these findings, our primary objective was to identify a diagnostically relevant Treg sub-population in patients with diverse autoimmune and inflammatory (AI/I) diseases, with or without underlying genetic defects, that could be applicable in a clinical immunology laboratory setting. We also aimed to determine the integrated immunological markers by analyzing immune cell subsets, CD19^+^ B, CD16/56^+^ NK, CD8^+^ cytotoxic T cells, and diversity of autoantibodies, which are prominent indicators of AI/I diseases. Therefore, we aimed to find integrated markers for the progression of AI/I diseases, independent of underlying mechanisms and distinct organ involvement by a holistic approach.

## 2. Patients and Methods

### 2.1. Study Population

After obtaining written informed consent for this study, patients with at least one AI/I condition, with or without IEI, were examined among those who had either newly consulted or were being followed up at the pediatric immunology department of a tertiary university hospital between 2019 and 2021. The patients with at least one AI/I finding, with or without inborn errors of immunity, were included in our study. The patients with allergy and malignancy were excluded from the study.

Demographic and laboratory data, including lymphocyte counts and autoantibodies, were collected from patient files. AI/I diseases were classified according to organ involvement, such as rheumatologic, neurologic, dermatologic, hemotologic, and endocrinologic.

The patient’s basic laboratory tests were reviewed for the presence of autoantibodies such as ANA (Anti-Nuclear Antibody), ENA (Extractable Nuclear Antigen), anti-dsDNA (Anti-double stranded DNA), antithyroglobulin, and anti-TPO (anti-thyroid peroxidase), and the results are noted in Table 1.

### 2.2. Flow Cytometry

Peripheral blood samples were collected from patients and control individuals. Peripheral blood mononuclear cells were obtained by using the density gradient method. Following the Buffy coat’s collection to a fresh tube, cells were washed once using Phosphate Buffer Saline (PBS) and centrifuged at 300× *g* for 10 min. The supernatant was removed, and the pellet was resuspended in 1× PBS (Thermofischer Scientific, Waltham, MA, USA). A 100 µL cell suspension was transferred to a flow cytometry tube, and anti-CD4, anti-CD127, and anti-CD25 antibodies were added for 30 min at room temperature and dark conditions (BD, Franklin Lakes, NJ, USA). Following incubation, cells were washed with 1× PBS and centrifuged. After removing the supernatant, a commercial 1× fixation buffer was added to the cell pellet for fixation for 10 min (BD, USA). Following the washing step, the cells were permeabilized with a commercial permeabilization buffer for 30 min (BD, USA). Then, cells were washed, and anti-FOXP3 antibody was added for 30 min in dark conditions (BD, USA).

For lymphocyte subset analyses, 100 µL of whole blood was stained with anti-CD19, anti-CD8, and anti-16/56 antibodies for 30 min. Cells were lysed with commercial lysing buffer for 10 min and washed with PBS, and centrifuged. Resuspended cells were analyzed at the FACS CANTO II flow cytometer (BD, USA).

### 2.3. Statistical Analysis

The statistical analyses were performed using SPSS software version 18 (New York, NY, USA) and R version 4.3.1. To compare the ratio of the Treg subsets and specific Treg markers (CD127, CD25, and FOXP3 expressions) between the patients and the healthy controls, the Mann-Whitney U test (Wilcoxon rank-sum test) was used. *p* < 0.05 was evaluated as significant. While investigating the associations between variables, Pearson’s and Spearman correlation coefficients and their significance were calculated for the assessment of the correlation between parametric and non-parametric variables, respectively.

R version 4.3.1 was utilized to implement a series of multivariate and machine learning techniques to investigate immunophenotypic heterogeneity among patients with immunological dysregulation. Correlation-based network visualization, supervised classification (random forest), unsupervised clustering (k-means), dimensionality reduction (t-distributed stochastic neighbor embedding, t-SNE), and receiver operating characteristic (ROC) analysis were all included in the analysis.

The spatial separation of healthy controls and patients was rendered feasible by using t-SNE to visualize high-dimensional flow cytometry data based on the percentage of important immunological markers (FOXP3, CD25, CD127, CD8, CD19, and autoantibody counts). Patients with immunological dysregulation were divided into data-driven immune subgroups using k-means clustering. The Elbow approach was used to determine the ideal number of clusters.

The relative significance of immunological markers in differentiating patients from healthy controls was evaluated using random forest analysis. The mean decrease in Gini impurity was used to rank the variables’ relative relevance. To assess diagnostic performance, ROC analysis was performed for each marker, and the pROC software v1.18.5 was used to compute area under the curve (AUC) values.

Spearman’s rank correlation coefficients were used to create correlation matrices. A simple correlation network was created by using a correlation threshold (|ρ| > 0.3). The immunological profiles of the discovered patient clusters were visualized and compared using radar plots, which displayed multidimensional variation.

The ggplot2, ggpubr, pheatmap, qgraph, and fmsb packages were used to create each plot. Unless otherwise indicated, complete-case analysis was used to address missing data.

## 3. Results

### 3.1. Patients’ Characteristics

**Figure 1:**
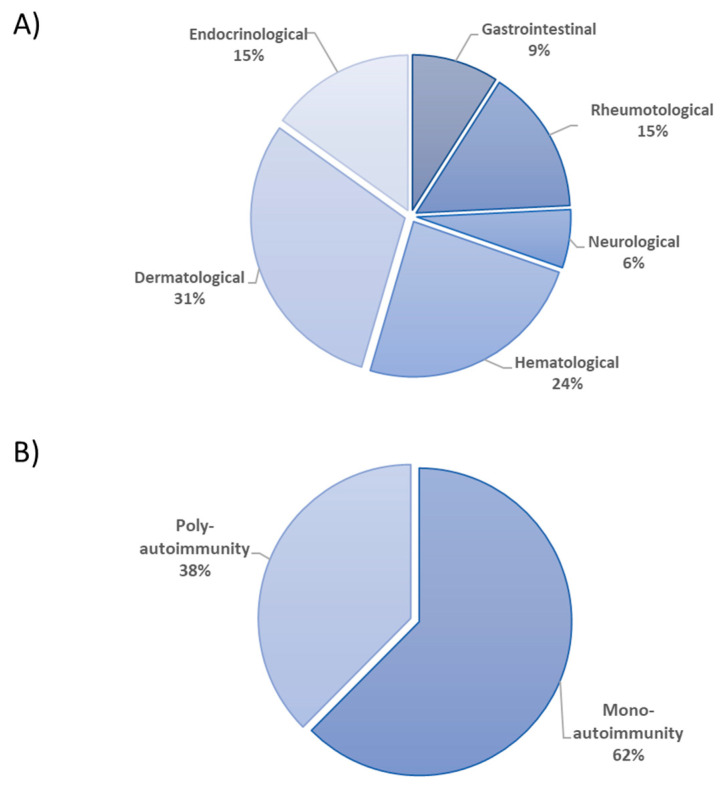
(**A**) The ratio of the organ systems affected by AI/I disorders. (**B**) The percentages of mono or multiple AI/I diseases among all investigated patients.

Forty patients with AI/I, accompanying with or without IEIs, were included in this study. The age ranges of the patients were between 8 and 492 months (Median = 104.5). The female to male ratio was 1/2. The age range of healthy controls was 288-408 months, and the median age of the control individuals was 360 months. The control male/female ratio was 7/12. Five patients had gastrointestinal AI/I disease, including inflammatory bowel disease (IBD) and autoimmune hepatitis, when examined according to the affected organ systems (9%). Rheumatologic diseases (15%) observed in the patients included Systemic lupus erythematosus (SLE) and Juvenile idiopathic arthritis (JIA). Neurological disorders (6%) which were observed in the patients included Multiple Sclerosis (MS), Myasthenia gravis, and Fuchs heterochromic iridocyclitis (FHI). Hematological disorders (24%) were idiopathic thrombocytic purpura (ITP), autoimmune hemolytic anemia, autoimmune neutropenia, aplastic anemia, and Castleman disease. Atopic dermatitis, psoriasis, and vitiligo were dermatologic autoimmune disorders shown in ten patients (31%) (Figure 1A). Twenty-five patients had distinct mono-autoimmune diseases (62%) and fifteen patients had multiple autoimmune manifestations (38%) (Figure 1B). Ten patients had AI/I manifestations accompanied by IEI (Table 1). Mutational analyses showed that LRBA gene defects were found in five patients. Other mutations were seen in *HAX1, STAT1, IL10RA, DOCK8*, and *ADA2* genes in patients with IEIs (Table 1).

### 3.2. Analysis of Treg Cell Sub-Populations

**Figure 2:**
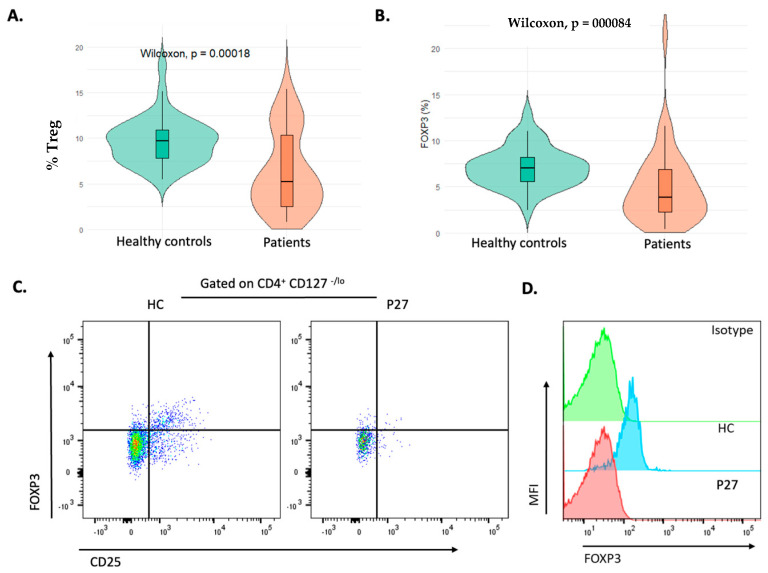
(**A**,**B**) Comparison of CD4^+^CD127^lo^ CD25^hi^ FOXP3^+^ Tregs and FOXP3 expressions in the patients and healthy controls. (**C**) Gating strategy of CD4^+^CD127^lo^ CD25^hi^ FOXP3^+^Tregs. (**D**) FOXP3 expression level in P27 and the healthy control.

During analysis of Treg cell populations, lymphocytes were separated according to FSC, SSC characteristics. Then, Treg cell sub-populations were separated at the CD3^+^ CD4^+^ lymphocyte gate. The median value of CD4^+^CD127^lo^ CD25^hi^ FOXP3^+^ Tregs was 4.4% (min = 0.8; max = 15.4) in the patients. The median value of these cells for control individuals was 8.1% (min = 4.8; max = 18). The Treg ratio was significantly lower in healthy control subjects (*p* < 0.001) (Figure 2A). Moreover, significantly decreased levels of FOXP3 expression were observed in the patients compared to healthy controls (Figure 2B). P27 had severely decreased level of CD4^+^CD127^lo^ CD25^hi^ FOXP3^+^ percentage and FOXP3 expression (Figure 2C,D). The ratio of CD4^+^ CD25^+^ CD127^lo^, CD4^+^ CD127^lo^ FOXP3^+^ and CD4^+^CD25^hi^FOXP3^+^ Treg sub-populations did not show significant change in the patients compared to healthy controls. In the patients and the healthy controls, the median values for CD4^+^ CD25^+^ CD127^lo^ Tregs were 17.1% (min = 0.4 and max = 48) and 16.55% (min = 6.6 and max = 35.6), respectively. In CD4^+^ CD127^lo^ FOXP3^+^ sub-population of Tregs, the median ratio was 63.6% in the patients. In the healthy controls, the median ratio was 40.1%. The median value of CD4^+^CD25^hi^FOXP3^+^ Treg sub-population was 4.2 in the patients (min = 0.2; max = 8.4).

### 3.3. Multidimensional and Machine Learning-Based Analysis

**Figure 3:**
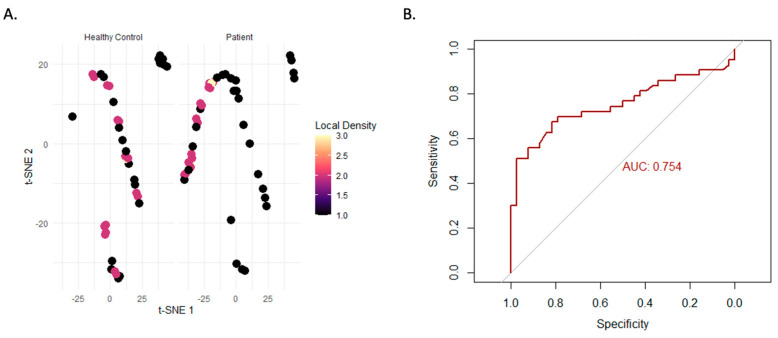
t-SNE visualization and ROC analysis of FOXP3 expression in Treg cells. A. The distribution of FOXP3+ regulatory T cells (Tregs) in patients and healthy controls is displayed in the t-Distributed Stochastic Neighbor Embedding (t-SNE) plot. (**A**) The cell is represented by each point, which is colored according to the local cell density; higher local density is shown by warmer colors. Labels are applied to clusters that relate to patients and healthy controls (**B**). Receiver operating characteristic (ROC) curve of FOXP3, which is utilized to differentiate Tregs between healthy control and the patients.

In this study, we used a multi-step strategy that included unsupervised clustering and statistical comparisons to identify unique immunological phenotypes among patients with clinically diagnosed immune dysregulation. The patient group’s t-SNE projection of FOXP3^+^ cells revealed a scattered and inconsistent spatial distribution, whereas the control group’s profile was more compact compared to other Treg markers such as CD25 and CD127 expressions (Figure 3A). In support of this finding, ROC analysis showed that evaluation of FOXP3 expression had good diagnostic value, discriminating patients from controls with an AUC of 0.754. Taken together, these findings suggest that FOXP3 may be a useful biomarker for immunological dysregulation (Figure 3B).

### 3.4. Data-Driven Immune Clusters in the Patients Show Different Characteristics

**Figure 4:**
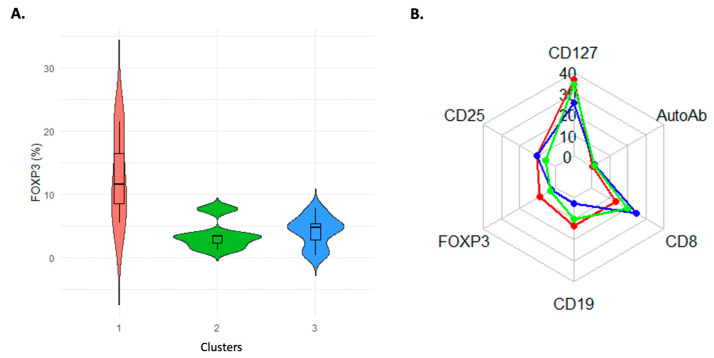
Comparative analysis of FOXP3 expression and immunophenotypic features in study groups. (**A**) The distribution of FOXP3^+^ cell percentages among the three groups is displayed in violin plots. Healthy controls (red) constitute Group 1, autoantibody-free patients (green) comprise Group 2, and autoantibody-positive patients (blue) form Group 3. The violins’ box plots show the interquartile range and median. Both patient groups show an apparent reduction in FOXP3 expression when compared to healthy controls (**B**). Mean marker expression levels in the same three groups are compared using a radar plot. Markers include CD127, Autoantibodies (AutoAb), CD8, CD19, FOXP3, and CD25. The lines that are red, green, and blue represent Groups 1, 2, and 3, respectively, emphasizing the different immunophenotypic characteristics of each group.

Three separate clusters were found within the patient cohort using unsupervised k-means clustering of the levels of FOXP3, CD25, CD127, CD8, CD19, and autoantibodies. Cluster 1 had high FOXP3 expression and low autoantibody levels, Cluster 2 had the lowest FOXP3 expression and the largest autoantibody burden, and Cluster 3 had an intermediate profile, according to violin plot analysis, which showed significant differences in FOXP3 expression between clusters (*p* < 0.01) (Figure 4A). Intra-group immunophenotypic heterogeneity was highlighted by the radar map display, which showed distinct multidimensional immunological signatures for every cluster (Figure 4B).

### 3.5. Correlations of Immunologic Markers with Treg Percentages

**Figure 5:**
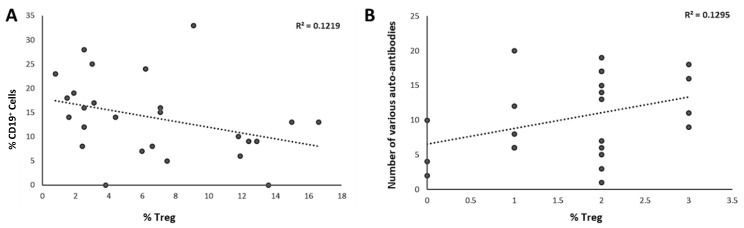
Correlation of CD4^+^CD127^lo^ CD25^hi^ FOXP3^+^ Treg sub-population with (**A**). CD19^+^ B cells. (**B**) The diversity of various autoantibodies.

We analyzed the correlation between the percentages of CD4^+^CD127^lo^ CD25^hi^ FOXP3^+^Tregs and CD8^+^ cytolytic T lymphocytes or CD19^+^ B lymphocytes, CD16/56^+^ NK cells. We did not find a correlation between CD4^+^CD127^lo^ CD25^hi^ FOXP3^+^Tregs and CD8^+^ or CD16/56 cell percentages. However, there was a negative correlation of CD4^+^CD127^lo^ CD25^hi^ FOXP3^+^Treg percentage with CD19^+^ B lymphocytes (Figure 5A). Moreover, we found a positive correlation between the diversity of autoantibodies and Treg percentages in the patients (Figure 5B).

## 4. Discussion

The immune system is specialized to protect against foreign and self-antigens, such as tumor antigens. However, in cases where the immune system’s response is impaired, either unresponsiveness and/or an excessive response of one or more components of the immune system to eliminate foreign antigens causes diseases. While inborn errors of immunity (IEIs) occur in the first scenario, in the latter, autoimmune or inflammatory diseases eventuate [7,8,38]. Different autoimmune manifestations that affect one or more organ systems coexist with immune dysregulation that are classified under IEIs [39,40,41,42,43,44,45,46,47,48]. Alterations in number/ratio and functions of Tregs, which are responsible for the immune tolerance, are characterized depending upon either primary defects that directly affect the development and function of Treg cells, such as in IEIs, or secondary effects such as in AI/I diseases. Treg cell counts/ratios are known to be variable depending upon the severity of the AI/I diseases. Primary defects of FOXP3 and CD25, which directly affect Treg cell development and function, cause IPEX (Immune dysregulation, poly endocrinopathy, enteropathy, X-linked) and IPEX-like disease, respectively [22]. Overlapping autoimmune manifestations are observed, such as severe enteropathy, diabetes mellitus, thyroiditis, and autoimmune cytopenias in these diseases [44]. Although some patients had normal FOXP3 expression and normal Treg ratios, several Treg cells were identified to be reduced or lost in addition to disrupted functional activity of existing Tregs in most of the FOXP3-deficient patients. Normal or slightly decreased Treg cell numbers are also observed in CD25 deficiency due to its crucial role in Treg development [44].

The CD4^+^CD25^+^FOXP3^+^ Treg subset was completely lost in three patients with IPEX; however, CD4^+^CD25^+^CD127^low^ Treg cells were comparable to the healthy donors. This subset was shown to exhibit compensatory suppressive function in these patients. However, this suppressive function was less than that shown by Treg cells from healthy controls. It is emphasized that FOXP3 expression is required for complete response of the CD4^+^CD25^+^CD127^low^ subset in these patients [45]. Although we did not find statistically significant alterations in the ratio of CD4^+^CD25^+^CD127^lo^, CD4^+^CD127^lo^ FOXP3^+^, and CD4^+^CD25^hi^FOXP3^+^ subsets, we demonstrated statistically significant change in CD4^+^CD127^lo^ CD25^hi^ FOXP3^+^ Tregs compared to healthy control subjects in our study. In children with autoimmune thyroid diseases, the CD4^+^ CD25^hi^ CD127^low^ FOXP3^+^ sub-population was shown to be significantly reduced compared to healthy donors, while there was no significant change in the CD4^+^ CD25^hi^ CD127^low^ sub-population [27]. Leading authorities in the field concluded that the CD4^+^ CD25^hi^ CD127^low^ FOXP3^+^ cells were the most suitable Treg sub-population for the assessment of clinical samples [48]. These findings demonstrated that during evaluation of the Treg cells, the CD4^+^ CD25^hi^ CD127^low^ FOXP3^+^ sub-population was more accurate than other sub-populations due to CD25 and CD127 expression levels together with FOXP3 expression. Depending on the investigated marker and the duration of the diseases, a differential proportion of Tregs was observed in these studies.

Furthermore, we showed significantly decreased FOXP3 expression as a good prognostic marker in the patients. Sustained expression of FOXP3 in Treg cells is critical for maintaining its suppressive capacity in life-threatening autoimmunity [46]. Numerous other candidate regulatory molecules, such as Lag-3, GITR, Helios, and Neuropilin, were initially considered as Treg-specific markers but were later dismissed due to lack of specificity. Numerous Treg phenotypes have been revealed by multiparametric analysis, and neither individual markers nor their combinations can fully characterize Treg. However, according to our study, the best combination of markers depends on the inflammatory process, independent of the system involved. In this respect, when examining immune dysregulation, it is important to evaluate the CD4^+^ CD25^hi^ CD127^low^ FOXP3^+^ subset as a predictive marker.

Similarly, the diseases with prominent autoimmune disorders such as LRBA and CTLA-4 deficiencies are determined as IPEX-like diseases and, owing to their convergent manifestations and dysfunctions, as well as altered numbers of Tregs [5]. LRBA, CTLA-4, DEF6, and STAT3 (GOF) deficiencies are currently classified under regulatory T cell defects [45]. A decreased proportion of Treg cells was associated with accompanying chronic inflammation and autoimmune findings in patients with CVID [40]. Besides, altered ratio of distinct Treg sub-populations was observed in most autoimmune diseases such as autoimmune hepatitis, multiple sclerosis, atopic dermatitis, and autoimmune thyroid diseases [40,41,42,43,44,45,46,47,48]. Although there is no consensus for Treg markers in the evaluation of patients’ samples, our multidimensional analysis showed that FOXP3 expression is a reliable indicator for differentiating patients with immunological dysregulation from healthy controls. Conversely, CD25 expression did not show any diagnostic significance and did not differ substantially between groups. CD25, despite being a classical Treg marker, may not have discriminatory specificity in immunological environments that are heterogeneous. These results further highlight FOXP3^+^ Tregs’ potential use in biomarker-guided diagnostics and align with previous findings of decreased levels of these cells in autoimmune or inflammatory disorders, such as atopic dermatitis [28,29]. These results further highlight FOXP3^+^ Tregs’ potential use in biomarker-guided diagnostics and are consistent with previous research showing decreased levels of these cells in patients with autoimmune or inflammatory disorders such as atopic dermatitis 

Using specific descriptive markers in the evaluation of Tregs in AI/I diseases is significant due to the perplexing feature of Treg cell plasticity, which is regulated by exogenous and endogenous conditions [23]. Due to the ongoing or intermittent release of tissue-specific factors governed by self- or infection-specific antigens and epigenetic control of FOXP3 during chronic inflammatory processes of autoimmune disease, Treg cells may acquire a conventional phenotype [18]. These conditions are also related to the limited precision of the phenotype of Treg sub-populations. Timing, the presence of an infectious agent, and treatment during the disease’s progression are also crucial factors for the investigation of Treg cell sub-populations with their unique cell surface and intracellular markers. Therefore, we excluded the possibility that infection history affected Treg ratios, as only six patients had acute or persistent infections, and did not show reduced Treg sub-populations. Furthermore, we are aware that immune suppressive treatments may affect the Treg ratio in patients, but only three patients in our cohort were under immune suppressive treatment during the study.

Crucially, in our study, three immunological subgroups with unique FOXP3-centered immune signatures were identified among patients using unsupervised k-means clustering. According to these data-driven clusters, immune dysregulation is not a single condition but rather a range of phenotypes with possible clinical significance. They are (1) high FOXP3 and low autoantibody load, (2) low FOXP3 and high CD8/autoantibody expression, and (3) intermediate patterns. Positive correlation between diversity of autoantibodies and the ratio of CD4^+^CD127^lo^ CD25^hi^ FOXP3^+^ Treg cells was found in the patients. We also demonstrated a negative correlation between CD19^+^ B cells and CD4^+^CD127^lo^ CD25^hi^ FOXP3^+^ Treg cells. This is not surprising due to Treg cells mediating B cell apoptosis and suppressing B cell function via cell-cell contact or cytokines such as TGF-β and IL-10 [49]. Treg cells have roles in B cell responses and B cell-mediated antibody production. The functional importance of these cells has been elucidated through studies of IPEX syndrome (immune dysregulation, polyendocrinopathy, enteropathy, X-linked), a disorder characterized by a profound deficiency in their number. In the sera of IPEX patients, numerous and varied autoantibodies are frequently found, indicating that Tregs are an important regulator of autoreactive B cells. In IPEX syndrome, FOXP3 deficiency indirectly impairs B cell tolerance by disrupting Treg-mediated regulation of peripheral tolerance checkpoints. FOXP3 mutations lead to an increased frequency of autoreactive B cells, dysregulated IgA and IgE synthesis, and the early appearance of tissue-specific autoantibodies. This dysregulated function causes autoreactive B cell accumulation in the periphery and promotes the production of pathogenic autoantibodies, thereby aggravating clinical manifestations [50,51]. Our data support the idea that autoantibodies are involved in chronic inflammation processes, such as in AI/I diseases, which prompt Treg cells to compensate for the inflammation.

In conclusion, in our study, we aimed to determine easily accessible, low-cost, rapid, and holistic markers that can be readily implemented in clinical laboratories. Although the number of patients was limited, our cross-sectional study—including immunophenotyping and integrated data-driven methods—demonstrated the importance of the investigated Treg sub-populations and the FOXP3 expression levels, which emerged as a reliable marker, particularly when evaluated alongside B cell ratio and autoantibody diversity in assessing immune dysregulation.

## Figures and Tables

**Table 1:** Clinical and laboratory findings of the patients.

Patients’	Age	Sex	Clinical Diagnosis	Affected Gene	% CD4^+^CD127^lo^	FOXP3	Presence of Autoantibody
Number					CD25^hi^ FOXP3^+^	Expression	
P1	6 y 1 m	Female	IBD	*IL10RA* and *MEFV*	11.7	N	nd
P2	38 y	Female	IBD	*STAT1*	15.4	N	neg
P3	16 y 1 m	Male	SLE, Hemolytic anemia,	nd	6.4	N	Anti-dsDNA, anti-thyroglobulin,
			Hashimoto thyroiditis				ENA, anti-TPO
P4	1 y 11 m	Male	FHI	nd	6.6	N	Anti-thyroglobulin, anti-TPO
P5	30 y	Female	MS, CVID	nd	12.9	N	Anti-thyroglobulin, anti-TPO
P6	2 y 1 m	Male	Pustular psoriasis, Cow milk allergy	nd	9.1	N	nd
P7	33 y 11 m	Female	CVID, AIHA, SLE, Livedo vasculitis	nd	3.6	N	Anti-thyroglobulin, anti-TPO
P8	13 y 10 m	Male	Cytopenia	nd	15	N	Anti-thyroglobulin, anti-TPO
P9	8 y 10 m	Male	Ectodermal dysplasia	nd	11.9	N	Anti-thyroglobulin, anti-TPO
P10	3 y 7 m	Female	Type 1 DM, Atopic dermatitis	nd	4.4	N	neg
P11	1 y 8 m	Male	Atopic dermatitis	nd	12.4	N	nd
P12	13 y 7 m	Female	Castleman Disease, Paraneoplastic pemphigus,	nd	13.6	N	Anti-dsDNA, anti-thyroglobulin,
			Myasthenia gravis, SLE				anti-TPO
P13	12 y 5 m	Male	IBD, sclerosing cholangitis,	*DOCK8*	4.6	N	Anti-thyroglobulin, anti-TPO
			portal hypertension, FMF				
P14	14 y 10 m	Male	Aplastic anemia, hyperglycemia	nd	2.1	N	neg
P15	9 y 8 m	Male	Autoimmune neutropenia, MDS	nd	6.2	N	neg
P16	13 y	Male	JIA, CF	*MEFV*	11.8	N	neg
P17	10 y 1 m	Male	Atopic dermatitis	nd	7.5	N	Anti-thyroglobulin, anti-TPO
P18	7 y 4 m	Male	Vitiligo	nd	7.1	N	nd
P19	5 y 8 m	Male	Hypothyroidism, Tubulopathy,	nd	3.8	N	Anti-dsDNA, anti-thyroglobulin,
			Milk and egg allergy, IEI				ENA, anti-TPO
P20	16 y 6 m	Female	Autoimmune hypophysitis,	nd	6	N	Anti-thyroglobulin, anti-TPO
			Autoimmune polyglandular syndrome				
P21	1 y	Male	Autoinflammatory disease,	nd	2.4	N	Anti-thyroglobulin, anti-TPO
			Graft versus host disease, psoriasis				
P22	8 y 11 m	Male	Aplastic anemia	*ADA2*	2.5	N	Anti-thyroglobulin, anti-TPO
P23	8 m	Male	Atopic dermatitis, milk and egg allergy	nd	1.6	N	Anti-dsDNA
P24	8 y 7 m	Female	DM, Hashimoto’s thyroiditis	nd	2.5	N	Anti-thyroglobulin, anti-TPO
P25	3 y 2 m	Male	Neutropenia, hypogammaglobulinemia	*MPO*	1.9	N	neg
P26	10 m	Male	Atopic dermatitis	nd	0.8	Severely	nd
						decreased	
P27	1 y 6 m	Male	IPEX	nd	0.9	Severely	Anti-thyroglobulin, anti-TPO
						decreased	
P28	12 y 3 m	Female	IBD	nd	11	N	Anti-dsDNA
P29	14 y 1 m	Male	Type 1 DM	nd	4.1	N	Anti-thyroglobulin, anti-TPO
P30	3 y 10 m	Male	Hypogammaglobulinemia,	nd	2.3	N	nd
			resistant atopic dermatitis,				
			multiple nutrient allergies				
P31	17 y 11 m	Female	Congenital neutropenia	*HAX1*	4.7	N	Anti-TPO, anti-thyroglobulin
P32	14 y 7 m	Female	Recurrent ITP	*LRBA*	3.9	N	Anti-thyroglobulin, anti-TPO
P33	46 y	Female	SLE, AIHA, CVID	*LRBA*	4.8	N	neg
P34	12 y 7 m	Female	ITP, CVID	*LRBA*	5.2	N	nd
P35	6 y 4 m	Female	AIHA	*LRBA*	5.3	N	nd
P36	4 y 3 m	Male	Alopecia areata, vitiligo	nd	4.9	N	Anti-thyroglobulin, anti-TPO
P37	16 y 5 m	Male	Autoimmune hypothyroiditis,	nd	5.3	N	nd
			Alopecia areata, CVID				
P38	14 y 9 m	Male	AIHA, Crohn’s disease, Oligo JIA	*LRBA*	6.5	N	nd
P39	6 y 7 m	Male	JIA	nd	10.3	N	nd
P40	41 y	Male	Autoimmune hepatitis, sjogren’s syndrome primary	nd	11	N	Anti-thyroglobulin, ANA
			biliary cholangitis, PFO				

nd: not determined; N: normal; neg: negative.

## Data Availability

The data that support the findings of this study are available from the corresponding author upon reasonable request.
